# Gas Gangrene

**Published:** 1917-12

**Authors:** 


**Gas Gangrene. Its Course and Treatment.** Cont. of abs. from p. 102 of the article by Kenneth Taylor.

According to T. treatment for gaseous gangrene consists in :

1. Prophylactic treatment during the dormant stage.
2. Treatment during the stage of gaseous distention.
3. Treatment of accomplished gangrene.

*Prophylactic treatment during the Dormant Stage* includes: "First, an attempt toward the removal or destruction of the bacteria present in the wound and toward depriving them of their necessary soil, the dead muscle; second, the institution of precautionary measures against the occurrence of gaseous distention. Time is an important factor. The shorter the interval between injury and treatment, the more certain is a successful result. Thorough cleansing of the fresh wound is necessary, including the removal of all foreign bodies possible. (Under " foreign bodies " we include dirt, fragments of cloth, fragments of bone, and also any portions of muscle showing signs of necrosis.) Following the cleansing of the wound, the use of an antiseptic active against the gas bacillus is clearly indicated ". For this purpose T. recommends a 1/10 o/o solution of quinine hydrochloride in physiological saline as effective clinically. Quinine proved experimentally, *in vitro* and in animals, to be much more active against the organism than any other solutions tried.

T. has found that other solutions commonly used have little bactericidal power *in vitro*. Potassium permanganate and hydrogen peroxide, even when used in very large amounts, only temporarily inhibit the growth of the organism *in vitro*. The bacillus grows readily in a medium with as much as 2 o/o of carbolic acid or 50 o/o of Dakin's solution.

Oxygen, injected subcutaneously, cannot reach the seat of the infection in the muscle, and it probably only increases the difficulties of circulation and tends to give a wrong idea of the extent of the gas formed by the bacilli.

" The use of anti-sera and vaccines is of doubtful value, if we consider the organism as a saprophyte, which has not invaded living tissue, and the damage done to the tissues as of a mechanical nature. It is also very uncertain that the muscle-toxic, hemolytic principle formed by the bacteria is a true soluble exotoxin for which an antitoxic serum can be produced. "

" In order to avoid the accident of gaseous distension, the following points must be considered : The wound should be kept as widely open as possible by means of a loose pack of gauze soaked in some wet dressing, preferably the quinine solution mentioned above. Any form of bandage or spint which increases pressure in the neighborhood of the wound or blocks the escape of gas, such as tight circular plaster casts, and so forth, must be avoided. The circular bandage bound tightly about a limb containing a fresh wound is no doubt frequently responsible for the development of gas gangrene. Wherever possible a gauze pad should be substituted and fixed in position with adhesive tape instead of with a circular bandage. Where fractures of the long bone are present, the limb should be put in extension as soon as possible, in order to keep the wound wide open. These points are especially important during the period of transportation of the wounded man ".

*Treatment during the Stage of Gaseous Distension.* — This stage is marked by increasing intramuscular pressure, which may result in the speedy death of the muscle. The pressure should therefore be relieved at the earliest possible moment. It is necessary to practice more incisions for the release of gaseous pressure than are needed for the draining of exudates. It is highly important, if possible, to find the focus of necrotic tissue where the gas is being formed and to remove all necrotic portions. Post-operative treatment should be similar to that mentioned above.

*Treatment of Gangrene.* — " If incisions into the muscle show a pale, dry, dull pink surface, and a consistency as if wrung dry of blood and lymph, the condition of gangrene is probably accomplished. The dead muscle is then a great menace to the patient, first, because it will speedily become an active source of gas production by the rapid invasion of the bacilli, and secondly, because the products of autolysis of a large mass of tissue may of themselves produce a profound toxemia. Muscle in this condition will never regain its vitality. If the patient lives it will be found to slough out in large fragments, sometimes as an entire muscle. Hence the treatment indicated is to remove the gangrenous tissue as quickly and as thoroughly as possible. This can usually be done only by amputation, if the process is in an extremity. No attempt should be made to cover the stump with skin flaps. The transverse section of the muscle fibers allows of free drainage of gas, and, unless extensive necrosis has occurred in the muscle tissues remaining, the process is frequently checked. The presence of subcutaneous crepitus above the possible limit of amputation, or even the presence of muscle involvement above that line, does not mean that the process will continue after the operation. "

